# Fear and Exploration in European Starlings (*Sturnus vulgaris)*: A Comparison of Hand-Reared and Wild-Caught Birds

**DOI:** 10.1371/journal.pone.0019074

**Published:** 2011-04-15

**Authors:** Gesa Feenders, Kristel Klaus, Melissa Bateson

**Affiliations:** Centre for Behaviour and Evolution, Institute of Neuroscience, Newcastle University, Newcastle upon Tyne, United Kingdom; University of Lethbridge, Canada

## Abstract

The revision of EU legislation will ban the use of wild-caught animals in scientific procedures. This change is partially predicated on the assumption that captive-rearing produces animals with reduced fearfulness. Previously, we have shown that hand-reared starlings (*Sturnus vulgaris*) indeed exhibit reduced fear of humans compared to wild-caught conspecifics. Here, we asked whether this reduction in fear in hand-reared birds is limited to fear of humans or extends more generally to fear of novel environments and novel objects. Comparing 6–8 month old birds hand-reared in the lab with age-matched birds caught from the wild as fledged juveniles a minimum of 1 month previously, we examined the birds' initial reactions in a novel environment (a small cage) and found that wild-caught starlings were faster to initiate movement compared to the hand-reared birds. We interpret this difference as evidence for greater escape motivation in the wild-caught birds. In contrast, we found no differences between hand-reared and wild-caught birds when tested in novel object tests assumed to measure neophobia and exploratory behaviour. Moreover, we found no correlations between individual bird's responses in the different tests, supporting the idea that these measure different traits (e.g. fear and exploration). In summary, our data show that developmental origin affects one measure of response to novelty in young starlings, indicative of a difference in either fear or coping style in a stressful situation. Our data contribute to a growing literature demonstrating effects of early-life experience on later behaviour in a range of species. However, since we did not find consistent evidence for reduced fearfulness in hand-reared birds, we remain agnostic about the welfare benefits of hand-rearing as a method for sourcing wild birds for behavioural and physiological research.

## Introduction

Wild-caught, non-domesticated species, especially birds, are widely used in laboratory research as models for biological phenomena that cannot be studied in standard laboratory animals [1]. This is likely to become more difficult in future because pending changes in European legislation introduce a ban on the use of wild-caught animals in scientific procedures [2]. This change in legislation is partially predicated on the assumption that captive-reared animals exhibit reduced levels of fear of humans [3]. Since freedom from fear is regarded as a fundamental requirement for acceptable standards of animal welfare [4], changes in animal husbandry that reduce fear are desirable and should be implemented where possible. However, particularly in the wild avian species most likely to be affected by the ban, little evidence exists to support the assumption that captive-reared animals are less fearful. This lack of evidence is concerning, given the high costs of captive breeding/rearing and the potential for other, possibly undesirable, effects on the animals that could alter their value as models for behavioural and physiological research [5], [6], [7].

The European starling (*Sturnus vulgaris*) is one of the most commonly used passerine bird species in laboratory research [1] and possibly also the most commonly used wild vertebrate animal. Starlings cannot easily be bred in captivity [8] and researchers currently source this species by catching adult or fledged juvenile birds from the wild [9]. Given that captive breeding is not an option for starlings, hand-rearing chicks taken from wild nests prior to filial imprinting has been recommended as an alternative strategy for creating tamer birds [3] and is currently being promoted as best practice by some Home Office inspectors within the United Kingdom. A recent study in our lab demonstrated that compared with wild-caught conspecifics hand-reared starlings indeed show a reduced withdrawal response from a human entering the laboratory, a finding that we interpreted as evidence of reduced fear of humans [10]. In the current study we extended this work to ask whether the observed reduction in fear is specific to the birds' reaction towards humans, or whether hand-reared starlings exhibit a generally lower level of fear in other stressful situations not involving humans. Reduced fear of humans could arise as a result of specific habituation to human presence and handling, or alternatively, it could reflect a reprogramming of the animals' stress responsiveness that would affect the birds' physiological and behavioural reactions to a wide range of stressors (e.g., [11], [12]). Understanding the consequences of hand-rearing is clearly crucial for evaluating the costs and benefits of using hand-raised animals as subjects in behavioural research.

Fear is an adaptive psychophysiological response with the function of protecting the animal from potential danger through appropriate behaviour and thus enhancing survival [13], [14]. Fear has also been associated with behavioural inflexibility and therefore influences exploratory behaviour [15], [16], [17]. Although it is not clear how exactly fear and exploration are related [18], [19], [20], [21], [22], it is generally agreed that very high levels of fear inhibit all other motivational systems including exploration. It follows that the presence of exploratory behaviour can be taken as evidence of relatively low levels of fear/neophobia. For this reason, measures of exploration are often interpreted as providing information about fearfulness, and are the basis of some of the standard tests of fear (e.g.,[23]).

A handful of studies have investigated the impact of developmental history on measures of fear and exploration in captive birds. A reduced level of neophobia has been reported in captive-bred compared to wild-caught African Grey parrots (*Psittacus erithacus*) [24], and in hand-reared compared to parent-reared orange-winged Amazon parrots (*Amazona amazonica*) [25]. The latter effect however did not persist post-weaning when both groups of birds were subjected to the same environment without specific handling by a human. In addition, Meehan and Mench [26] showed that current housing conditions can have profound effects on exploratory behaviour: young Orange-winged Amazon parrots who had spent one year in enriched conditions showed reduced neophobia compared to those reared in a non-enriched environment.

Given the limited data described above, we set out to evaluate the effect of developmental history and environmental enrichment on fear and exploration in European starlings. We adopted an applied perspective and tested the birds under conditions a researcher would face if sourcing the birds either by catching independent juveniles or hand-rearing nestlings. We measured fear and exploration in two established tests, the Novel Environment Test (locomotor activity in a novel arena) and the Novel Object Test (reaction towards a novel object). In the latter test the novel object was either placed at a neutral location within the cage to elicit exploratory motivation as the bird was able to choose whether or not to approach the object, or at the food dish to elicit neophobia as the bird's motivation to approach the food is counteracted by neophobia towards the novel object [27], [28]. On the basis of previous findings, we predicted that hand-reared birds and birds housed in enriched cages would exhibit reduced levels of fear and thus increased exploration.

## Methods

### Ethics Statement

Our study adhered to the Association for the Study of Animal Behaviour's Guidelines for the Use of Animals in Research and also passed the Newcastle University Ethical Review Committee. The starlings were taken from the wild under Natural England licence number 20093194. The birds were released back into the aviaries after the experiment and retained for further studies.

### Subjects

A total of 32 European starlings, 16 wild-caught and 16 hand-reared (9 females and 7 males in each group) were used in the current study, but one wild-caught female died after having been in the cage for 7 days. In May 2009, hatchlings (6–12 days post-hatching, one bird per clutch) were taken from nest boxes in rural Northumberland, UK. The birds were hand-reared in the laboratory (14L:10D, ∼23°C) using a mix of soaked cat food and apple sauce, supplemented with vitamins and calcium. Once they became independent and started to feed for themselves (at around 3 weeks of age), they were transferred to an indoor aviary. The young birds were not exposed to adult starlings at any stage of the hand-rearing process (with the exception of some possible auditory contact from adjacent rooms). The wild-caught starlings were caught from the same population as the hand-reared birds in late September of the same year (i.e. at around 4 months of age), using a baited whoosh net. The breeding season of starlings in the North-East of England is short, allowing for only one brood, thus we could be confident that the wild-caught birds were of a similar age to the hand-reared birds.

When not being tested, hand-reared and wild-caught starlings were kept in separate indoor aviaries (3.60×2.40×2.25 m WDH) under a light-dark cycle of 14L:10D, at ∼18°C. Each aviary was enriched with ropes for perching, cardboard boxes for cover, and the floor was covered with bark chips to provide a natural substrate for the birds to probe in. The birds were provided with food (chick crumbs) and water ad libitum, supplemented with dried insect food (Orlux Insect Patee), fruit and mealworms. None of the birds had been in individual cages before the current test.

When tested (November 2009 – January 2010), all birds were approximately 6–8 months of age. Tests started after the wild-caught birds had been in captivity for 4 weeks (required quarantine period, including anti-parasite treatment every 10 days).

### Experimental cages

For testing, the birds were individually housed in cages (100×45×45 cm WDH), with wire mesh fronts and backs, solid sides, and a clear Plexiglas roof (for details, see Figure 1 in [10]). Eight cages were arranged on two shelf levels (38 and 120 cm height) in a single room such that all birds had visual contact with four to six of the other starlings in the room. Each cage was fitted with an overhead surveillance camera (Atom, CSP Technology, UK) connected to a computer in an adjacent room that was used for remote observation and video recording. For the first day and night, the cage tops were covered with paper to prevent the birds from injuring themselves by attempting to escape through the transparent Plexiglas ceiling. The covers were removed during husbandry on the following day. Husbandry was conducted daily at around 1200 hrs.

**Figure 1:**
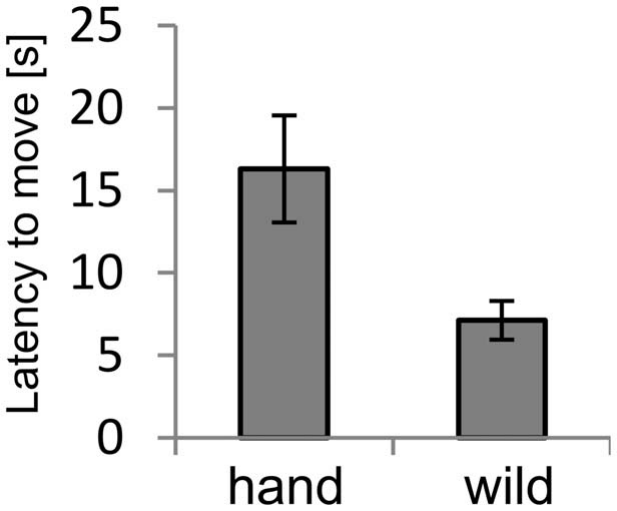
Behavioural responses of the birds in the Novel Environment Test. Shown is the latency to move. hand  =  hand-reared birds; wild  =  wild-caught birds. Data show group means ± 1 SEM.

Four cages were environmentally enriched with a plastic tray (18.5×13×6 cm WDH) filled with wood chips as a probing substrate, a water bath filled with water and a small plastic hide on the distal end of one of the two perches. The four non-enriched cages were furnished with an empty plastic tray and water bath (the bath was filled temporarily twice a week for one hour to ensure good hygiene).

Four replicate groups of eight birds were tested sequentially. The assignment of birds to cages was counterbalanced with respect to developmental history, enrichment condition and cage position. Each replicate took two weeks to complete, after which the birds were returned to their aviaries.

### Novel Environment Test

On day1 of testing, a group of eight birds were caught from the aviaries and placed in the cages while the room lights were switched off. After the last bird was put into its cage, the experimenter started video recording, left the room, and after one minute turned on the lights. This marked the beginning of the test which can be seen as a forced novel environment test because the bird was not placed in a start box from which it had to emerge, but in contrast was placed directly in the novel arena [29]. The birds were recorded for 30 min from the front with camcorders, as the cage tops were covered for habituation purposes. During the recording, no water baths were provided as they would have partly obstructed the view.

### Novel Object Test 1

During the week preceding the Novel Object Tests the birds were habituated to visual isolation from the other birds in the room by drawing white opaque curtains between the cages for one hour each morning. This visual isolation was used during the tests to prevent birds from being influenced by seeing other birds' reaction to the novel objects.

Novel Object Test 1 was carried out on day 8, day 9, and day 11. On each testing day at 60 min after the lights came on, the curtains were closed, the lights turned off and a novel object was hung inside on the front cage wall beside the perch. 1 min after the experimenters left the room, the lights were turned on marking the start of the test. The birds were recorded for 10 minutes after which the curtains were opened and the objects removed.

The novel objects used in the tests were chosen so that the birds were unlikely to have had direct contact with similar objects in the past. The objects included a bright green clothes peg (1.5–3.0×1×7 cm), an orange ball (4 cm diameter), and a yellow furry pad (ca 5×3×7 cm). All birds experienced the objects in the same order: first the bright green clothes peg, second the orange ball, and third the yellow furry pad.

### Novel Object Test 2

On the same day as the last Novel Object Test 1 (day 11), a baseline trial for Novel Object Test 2 took place. During regular husbandry at 1200 hrs, the plastic trays and water baths were removed from the cages and the birds were left unfed. Upon completion at 1230 hrs, the curtains were closed and the plastic tray, now containing five meal-worms, was placed in each cage; for the enriched cages this tray also contained the wood chip filling so that the worms could move into the filling. All birds were used to obtaining worms from these trays, and were generally eager to eat them. The test started as soon as the cage door was closed. After 10 minutes of recording, the curtains were opened and the birds were provided with their regular food and water baths. The same procedure was repeated the following day (day 12) except that this time a novel object (a pale green sponge with brown stripes; 6×3.5×8 cm) was placed in the middle of the tray together with the five meal-worms.

### Behavioural measures

Behavioural data were scored manually using The Observer XT 8.0 (Noldus Information Technology, Wageningen, Netherlands) by a single observer (KK) who was blind to the developmental origins of the birds.

Once the lights came on in the Novel Environment Test, we recorded the bird's latency to move (Lat(move)), with immobility defined as staying in one location without moving with both its feet, flying, ruffling the feathers, stretching wings, or wiping the bill, allowing only head movements; we could not exclude the latter because of the angle and resolution of the video image. To obtain a measure of general activity, every transition between distinct cage locations (the two perches, front, back and side walls, floor and tray) was recorded (PositionChanges). In addition, jumps at ceiling (CeilingJumps), the total duration on cage walls, (T(walls)), latency to fly to the cage walls (Lat(walls)), and the latency to feed (pecking at the food, Lat(feed)) were measured as potential indicators of fear/stress/escape motivation. If the bird did not feed or go to the cage walls within 30 minutes, it was assigned a ceiling value of 1800 s.

In Novel Object Test 1, the latency to start moving (Lat(move)) and the latency to peck at the object (Lat(peck)) were recorded. If the bird did not peck at the object within the first 10 minutes, it was assigned a ceiling value of 600 s.

In the Novel Object Test 2, for both the baseline and test condition, the latency to peck at the meal-worm during the first 10 minutes was recorded. If the bird did not peck at the worm during this time, it was assigned a ceiling value of 600 s.

### Data analysis

In the Novel Environment and Novel Object Test 1, the latency to move was subtracted from the latency to feed/walls/peck in order to remove individual differences in latency to move. The number of CeilingJumps was expressed as a rate per PositionChange to account for individual differences in activity levels. For each of the latency and duration measures, the resulting data values were bounded between 0 and the maximum (remaining) observation time. In order to obtain normally distributed data for statistical analysis we expressed the times as a proportion of the maximum observation time, followed by arcsine square-root transformation. In Novel Object Test 2, the latency to peck at the worm in the baseline condition was subtracted from the latency in the test condition to remove individual differences in motivation to feed not attributable to the presence of the object; in this case, the resulting difference was not transformed further.

General linear models (GLMs) with each of the measures described above as the dependent variable were performed. The independent variables used in the models were as follows. For the Novel Environment test, origin (hand-reared versus wild caught) was included as a between-subject factor, but current housing condition (non-enriched versus enriched) was excluded as environmental enrichment was unlikely to have had any significant effect at this early stage and because one enrichment component (the water bath) was not present during this test. For Novel Object Tests 1 and 2, origin, housing and their interaction, origin × housing, were included as between-subjects factors. In all GLMs, we additionally included replicate group (four levels) as a blocking factor but not its interactions, because group was deemed to be an arbitrarily assigned factor unlikely to have non-additive interactions with our main treatments [30]. In the case of the Novel Environment Test, for which we had several dependent variables, we applied a Bonferroni correction for multiple testing resulting in a reduction in the value of alpha for these tests from 0.05 to 0.008.

In order to explore further the data from the Novel Environment Test, we performed a Principal Component Analysis (PCA) to reduce the number of dependent variables. PCA was performed on the z-scores of the behavioural variables described above computed after data transformation (where applicable) to correct normality. We used orthogonal rotation to maximize differentiation of the measures. Principal components with an eigenvalue >1 were taken for further analysis with GLMs to explore the effect of origin.

In cases where the assumption of homogeneity of variance or normality was violated, non-parametric tests were performed as indicated.

To observe the relationships of variables between tests, Spearman rank correlation was used because in most cases the data did not meet the assumptions of normality (Shapiro-Wilk test p<0.05) despite data transformation. Cronbach's alpha was used to test the internal reliability of the responses in the three repetitions of Novel Object Test 1.

## Results

### Novel Environment Test

GLMs on the individual measures from this test - Lat(move), Lat(feed), Lat(walls), T(walls), CeilingJumps, and PositionChanges - revealed a significant effect of origin on Lat(move) with the wild-caught birds showing shorter latencies (Figure 1). There were no effects on any of the other measures. Statistics (F-ratios and p-values) for these analyses are summarized in Table 1. We found no significant effects of replicate group in any of the analyses (Supporting Figure S1A), providing evidence that the birds' response to the laboratory environment was not continuing to change during the months of testing. Performing a PCA with the above listed variables yielded two principal components with eigenvalues >1 (Table 2). Together, PC1 and PC2 accounted for 62% of total variance. PC1 was positively correlated (factor loadings >0.640) with the latency measures (latency to move, feed, walls), but not with T(walls), CeilingJumps, or PositionChanges (factor loadings <0.220). PC2 was mainly correlated (factor loadings >0.550) with PositionChanges, CeilingJumps, and Lat(feed), but showed small factor loadings with the other variables (factor loadings <0.400). A subsequent GLM on PC1 scores, with origin as a between-subjects factor (and replicate group as blocking factor), resulted in a significant effect of origin (*F*(1,27)  = 10.22, *P* = 0.004); for PC2 the assumptions of homogeneity and normality were violated so we performed a Mann-Whitney *U* test, which yielded a non-significant effect of origin (*U* = 122, *N_1_* = *N_2_* = 16, *P* = 0.821).

**Table 1:** GLM analyses of the birds' behaviour during the three tests.

	measure	Origin (*F*, *P*)	Housing (*F*, *P*)	origin x housing (*F*, *P*)
**Novel Environment Test**				
*df* = 1,27	Lat(move)	**12.35, 0.002**	na	na
	Lat(feed)	1.84, 0.187	na	na
	Lat(walls)	2.56, 0.121	na	na
	T(walls)	4.13, 0.052	na	na
	CeilingJumps	0.55, 0.465	na	na
	PositionChanges	0.11, 0.742	na	na
**Novel Object Test 1**				
*df* = 1,24	Lat(move)	**6.52, 0.017**	0.38, 0.545	0.47, 0.500
	Lat(peck)	2.49, 0.127	1.18, 0.289	0.00, 0.966
**Novel Object Test 2**				
*df* = 1,24	diff(LatPeck)	0.17, 0.682	0.97, 0.335	2.34, 0.139

Tested are inter-individual effects from origin and housing. Shown are the *F*- and *P*-values. Significant effects are highlighted in bold (after Bonferroni-correction for Novel Environment Test). na  =  factor not included in model.

**Table 2:** Results of Principal component analysis.

Measure	PC1	PC2
Lat(walls)	0.856	0.104
Lat(move)	0.818	−0.103
Lat(feed)	0.641	0.555
PositionChanges	−0.065	−0.855
CeilingJumps	−0.170	0.785
T(walls)	−0.216	−0.396

Results of the birds' behaviour during the Novel Environment Tests. Shown are the factor loadings after rotation, sorted by PC1 values.

### Novel Object Test 1

**Figure 2:**
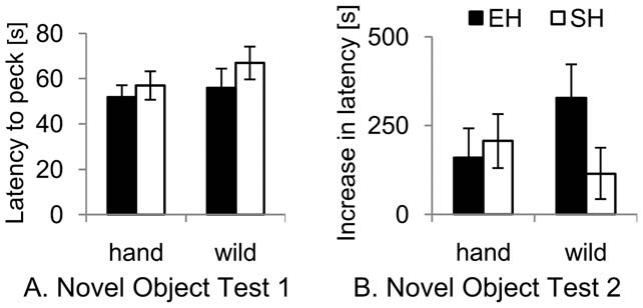
Behavioural responses of the birds in the Novel Object Tests. **A.** Novel Object Test 1: latency to peck the novel object; mean of three test repetitions. **B.** Novel Object Test 2: increase in latency to peck the food as a response to a novel object added to the food tray. hand  =  hand-reared birds; wild  =  wild-caught birds; EH (black bars)  =  enriched housing; SH (open bars)  =  non-enriched housing. Data show group means ± 1 SEM.

Over the three tests, only four birds (one hand-reared, three wild-caught) never pecked at any of the three objects. The birds were individually consistent across tests with respect to latency to move (Cronbach's alpha  =  0.670, *P*<0.001) and to peck at the object (Cronbach's alpha  =  0.595, *P* = 0.001). Based on this consistency in latency measures, the mean of the three tests was used in subsequent analyses to examine the effect of origin and housing on the responses. In accordance with the results from the Novel Environment Test, the wild-caught birds were again faster to start moving than the hand-reared birds. There was no difference between the two groups in their latency to peck at the novel object (Figure 2A, Table 1). No effect was observed for housing or the interaction origin × housing on either of the measures. Replicate group was significant (*P*<0.001) for Lat(move) in Novel Object Test 1. Supporting Figure S1B shows that the same changes over time are evident in both the hand-reared and wild-caught birds, arguing for a maturational change in both groups as opposed to continued habituation in the wild-caught birds.

### Novel Object Test 2

Although all birds pecked the worms in the control condition, a total of nine birds did not peck the worms in the test condition within 10 min (five hand-reared, four wild-caught). Overall, the birds were faster to peck at the worm in the control condition as compared to the test condition (Wilcoxon Signed Rank test, *Z* = −4.390, *N* = 31, *P*<0.001). The reduction in latency was not affected by origin, housing or their interaction (Figure 2B, Table 1).

### Correlations between tests

We found no correlations between the latencies to move in the Novel Environment and in Novel Object Test 1 (rho  =  0.013, *P* = 0.943), between Lat(peck) in Novel Object Test 1 and diff(LatPeck) in Novel Object Test 2 (rho  =  0.011, *P* = 0.952,), or between Lat(move) in the Novel Environment and diff(LatPeck) in Novel Object Test 2 (rho  =  0.161, *P* = 0.387).

## Discussion

In this study we examined the effect of developmental history and current housing conditions on fear and exploration in starlings. We found that hand-reared starlings were slower to start moving in a novel environment than wild-caught birds, but the response to novel objects was not different between the two groups. Contrary to expectations, environmental enrichment had no effect on any measure of behaviour in this study.

In the Novel Environment Test, developmental history affected the birds' latency to start moving when placed in novel cages for the first time, with the hand-reared starlings being slower than the wild-caught ones. This result from the General Linear Model is supported by the Principal Component Analysis showing that PC1, primarily correlated to the latencies (walls, move, feed), was affected by origin, while PC2 was not. Consistent with this, the latency to start moving was also different between the hand-reared and wild-caught birds in Novel Object Test 1.

Longer durations of immobility in similar tests have been associated with higher levels of fear [31], [32]. In our case, due to the camera position it was not possible to see the birds' head movements fully, thus we could not be certain about the duration of actual freezing behaviour. Instead, judging from the cases in which we had a good viewing angle, it seems most likely that the birds were freezing momentarily, followed by some head movements whilst otherwise sitting still. Given that PC1 was not only related to the latency to start moving but also strongly correlated with the latency to fly to the walls, a potential indicator of escape attempt [33], we suggest that the latency to move and latency to the walls might measure the same behavioural trait, i.e. escape motivation. Against this interpretation, the time spent on the walls and jumps to the ceiling, two different potential indicators of escape attempts, did not fall into the same principal component; on the contrary, jumps to the ceiling strongly contributed to the orthogonal PC2. However, both the time spent on the walls and jumps to the ceiling are cumulative measures, taken over the full observation period of 30 minutes, whereas the latencies to move and to the walls reflect the birds' initial response to the new situation, thus the former and latter measures could reflect different aspects of the birds' responses. In summary therefore, it seems plausible that the difference between the two groups in their latency to start moving was primarily driven by differences in initial escape motivation when the birds were first placed in the cages with the wild-caught birds showing higher escape motivation. It is not clear however what this means with regard to the relative affective states of the hand-reared and wild-caught birds. The difference in response might indicate that the hand-reared birds are less fearful than the wild-caught birds, but it could also indicate a difference in coping styles in stressful situations.

As we tested the birds at only one time point beginning just one month after the wild-caught birds were captured, it is possible that the differences between the hand-reared and wild-caught birds could be due to differences in habituation to the laboratory rather than developmental history. However, our data do not support the hypothesis that the wild-caught birds were still habituating to captivity during testing. The latency to move in the Novel Environment Test did not change across the four replicate groups, indicating no evidence for continuing habituation in the wild-caught birds over the 3-month period of testing (Supporting Figure S1A). Although we did find an effect of replicate group on the latencies to move in Novel Object Test 1, this effect was evident in both the hand-reared and wild-caught birds (Supporting Figure S1B), supporting an explanation based on maturation of the birds as opposed to differential habituation to the laboratory environment.

A second potential confound that could explain the difference between the two groups of birds is the method of catching. Personality differences (e.g. boldness) could have affected which birds were caught in the baited whoosh net [34], whereas there is no obvious mechanism by which personality could have affected our choice of chicks from the nests. However, if the wild-caught birds represent a more homogeneous sample than the hand-reared birds, as would be suggested by this hypothesis, then we would expect to see greater variation within the hand-reared group, and there is no evidence from any of our analyses to support this. Indeed, our observations suggest that on a number of measures the hand-reared birds showed less behavioural variation than the wild-caught group (unpublished results).

Despite the above evidence to the contrary, we cannot completely discount the possibility that the differences between the hand-reared and wild caught birds might be caused by differences in habituation or personality rather than our developmental manipulation. However, it is important to emphasise that from an applied perspective the actual mechanism underlying the differences is not important, because wild-caught birds will always have had less time in captivity than age-matched hand-reared birds, and wild-caught birds will always have been caught in some sort of trap. Thus habituation differences and personality differences are inevitably correlated with developmental origin. What is important from a scientific perspective is that young birds sourced in these different ways are likely to respond differently in some stressful situations.

We found no effect of origin on the neophobic response to a novel object attached to the food tray. This may support the idea that fear is not a singular trait that can be generalized across different situations [26], [35]. Alternatively, as has been demonstrated in previous studies, the two tests (Novel Environment and Novel Object Tests) may have induced different levels of fear yielding different behavioural responses. Previous studies have emphasized the important role of refuge access in rodents in the novel environment test: rats quickly entered a small darkened box [36], [37] or did not even leave the refuge when provided with a start box [38], [39], and when given a choice between entering a novel area or a home cage, the rats chose the home cage more frequently, even when having to confront a foot shock to do so [38]. In mice, behavioural responses were reflected in the plasma corticosterone levels that increased when the animals were placed in a novel environment without escape option, but remained unchanged when the animals were presented with a novel object in a familiar environment [29]. The authors of the latter study concluded that intense fear is only elicited when the animals are prevented from performing their natural behaviour in response to fear. On this basis, we argue that our “forced” Novel Environment Test was probably strongly fear-eliciting because the birds were unable to escape [29], [40], [41]. In contrast, in Novel Object Test 2 the starlings were free to choose whether or not to approach the tray. It therefore seems that the difference of fear response caused by developmental history only becomes apparent in situations of high fear or where there is no avoidance/escape option.

In Novel Object Test 1, we found no effect of the birds' origin on the latency to peck. This cannot be attributed to a lack of interest by the birds in the objects, since 27 of the 31 birds pecked at the objects once or more over the three tests, indicating at least some level of exploratory motivation. This result seems to contrast with previous findings from mammalian species showing increased exploratory behaviour in early handled animals [5], [42], [43], [44], [45]. Importantly, though, in other studies, laboratory-bred rodents showed lower exploratory behaviour than wild conspecifics [46], [47]. It seems likely that the beneficial effect of early handling might be partially offset by a negative effect of hand-rearing. The latter involves maternal deprivation which has been shown to have effects opposite to early handling on later stress responses [48]. Environmental enrichment has been shown to compensate for early maternal separation in rats [49], and to alleviate the effects of early traumatizing experience in parrots [24]. Thus, it is possible that the housing conditions of our hand-reared birds post-fledging were sufficiently stimulating to compensate for negative effects of the hand-rearing procedure. It is also possible that any effects of early handling may have diminished during the 4 months of independence prior to testing when the birds no longer experienced any significant handling (see [25]).

A number of previous studies have compared passerine birds' responses to novel environment and novel object tests with the aim of detecting stable individual differences in behaviour indicative of personalities or behavioural syndromes [27], [50]. The exploratory reactions of individual great tits (*Parus major*) to a novel environment and novel object are positively correlated, suggesting the existence of a personality trait of slow and fast exploration [27], [51]. In contrast, exploration and neophobia do not seem to be correlated in blue tits (*Cyanistes caeruleus*) [52] or garden warblers (*Sylvia borin*) [53] but the latter study found a negative correlation in Sardinian warblers (*Sylvia melanocephala*); authors of these latter two studies argue that the tests used may probe for independent personality dimensions (e.g. anxiety and boldness). In our data, we did not find any correlation between measures from different tests. Even the latency to move was not correlated between the Novel Environment Test and Novel Object Test 1 despite a significant effect of origin on both measures, and within-subject reliability within the repetitions of Novel Object Test 1. It seems that the birds were not consistent across context (unfamiliar environment versus familiar environment but with novel object), but the group-specific response profile was consistent. Thus, on one hand, the reliability of our birds during the three repetitions of Novel Object Test 1 suggests that the measures taken reflect stable individual differences (but see [54]). On the other hand, the lack of correlation between different tests supports Greenberg and Mettke-Hofmann's [18] idea of separate motivational systems for fear and exploration (see also [55]). Consequently, we argue that the latency to start moving in Novel Object Test 1 is a mix of those two motivations, with the fear response causing the difference between hand-reared and wild-caught birds, but this difference being further modified by exploratory motivation. In other words, in the Novel Environment Test fear may have been the primary motivation suppressing any exploration, resulting in different responses of the two groups, whereas in Novel Object Test 1 the response was driven by both motivations, resulting in a group difference (due to the different fear response) but no within-subject consistency due to individual differences in motivation to explore.

Throughout all tests we did not observe any effect of the current housing condition. This lack of effect of environmental enrichment could have derived from too short an exposure time, but we do not believe this to be the case because we have previously seen effects of environmental enrichment after only one week [56]. However, in that study more natural enrichments were used such as bark chips on the floor and natural perches. Thus, we suggest that in our current study the type of enrichment was not sufficiently different from the non-enriched condition to produce significant effects.

In conclusion, we found that 6–8 month old hand-reared starlings showed longer latencies to start moving in a novel environment than wild-caught birds of the same age. We interpret this difference as indicative of reduced escape motivation in the hand-reared birds. This finding confirms that hand-reared and wild-caught starlings differ in more respects than just their response to humans, and suggests that hand-rearing could result in a general alteration in how the birds respond to certain stressors. Our results add to the growing literature showing lasting effects of early-life events in a range of species. Thus from a scientific perspective, the developmental origin of European starlings could potentially affect the type of results obtained in experiments where the birds' response to a novel environment or other highly stressful situation could be a factor. From a welfare perspective, our results are less clear-cut, because using three established tests we found ambiguous evidence for a decrease in general fearfulness in hand-reared birds. We therefore remain agnostic about the welfare benefits of hand-rearing as a method for sourcing wild birds for behavioural and physiological research. Modest evidence for reduced fearfulness needs to be set against the longer periods of time hand-reared birds must spend in captivity and the problems releasing them to the wild at the end of a study.

## Supporting Information

Figure S1 **Effect of replicate group.** Effect of origin (hand  =  hand-reared; wild  =  wild-caught) and replicate group (different colours indicate replicate groups 1 to 4) on latency to move in (**A**) Novel Environment Test and (**B**) Novel Object Test 1. Data show group means ± 1 SEM.
(PDF)

## References

[1] Bateson M, Feenders G (2010). The use of passerine bird species in laboratory research: implications of basic biology for husbandry and welfare.. ILAR Journal.

[2] Directive of the European Parliament and of the Council on the protection of animals used for scientificpurposes..

[3] Hawkins P, Bairlein F, Duncan I, Fluegge C, Francis R (2003). Future principles for housing and care of laboratory birds: Report for the revision of the Council of Europe convention ETS123 appendix A for birds..

[4] Second report on priorities for research and development in farm animal welfare..

[5] Pryce CR, Feldon J (2003). Long-term neurobehavioural impact of the postnatal environment in rats: manipulations, effects and mediating mechanisms.. Neuroscience & Biobehavioral Reviews.

[6] Anisman H, Zaharia MD, Meaney MJ, Merali, Zul (1998). Do early-life events permanently alter behavioral and hormonal responses to stressors?. International Journal of Developmental Neuroscience.

[7] Lewis MH (2004). Environmental complexity and central nervous system development and function.. Mental Retardation and Developmental Disabilities Research Reviews.

[8] Bateson M, Asher L, Hubrecht R, Kirkwood J (2010). The European starling.. The UFAW Handbook on The Care and Management of Laboratory and Other Research Animals.

[9] Asher L, Bateson M (2008). Use and husbandry of captive European starlings (Sturnus vulgaris) in scientific research: a review of current practice.. Laboratory Animals.

[10] Feenders G, Bateson M (2011). Hand-Rearing Reduces Fear of Humans in European Starlings, *Sturnus vulgaris*.. PLoS ONE.

[11] Plotsky PM, Meaney MJ (1993). Early, postnatal experience alters hypothalamic corticotropin-releasing factor (CRF) mRNA, median eminence CRF content and stress-induced release in adult rats.. Brain Res Mol Brain Res.

[12] Sanchez MM, Ladd CO, Plotsky PM (2001). Early adverse experience as a developmental risk factor for later psychopathology: Evidence from rodent and primate models.. Development and Psychopathology.

[13] Jones RB (1986). Conspecific vocalization, tonic immobility and fearfulness in the domestic-fowl.. Behavioural Processes.

[14] Murphy LB (1978). The practical problems of recognizing and measuring fear and exploration behaviour in the domestic fowl.. Animal Behaviour.

[15] Greenberg R (1984). Differences in feeding neophobia in the tropical migrant wood warblers Dendroica castanea and D. pensylvanica.. Journal of Comparative Psychology.

[16] Martin LB, Fitzgerald L (2005). A taste for novelty in invading house sparrows, Passer domesticus.. Behavioral Ecology.

[17] Mettke-Hofmann C, Winkler H, Leisler B (2002). The significance of ecological factors for exploration and neophobia in parrots.. Ethology.

[18] Greenberg R, Mettke-Hofmann C (2001). Ecological aspects of neophobia and neophilia in birds.. Current Ornithology, Vol 16.

[19] Halliday MS, Jewell PA, Loizos C (1966). Exploration and fear in the rat.. Play, exploration and territory in mammals.

[20] Lester D (1968). Effect of fear and anxiety on exploration and curiosity - toward a theory of exploration.. Journal of General Psychology.

[21] Montgomery KC (1955). The relation between fear induced by novel stimulation and exploratory drive.. Journal of Comparative and Physiological Psychology.

[22] Russell PA (1973). Relationships between exploratory behavior and fear - review.. British Journal of Psychology.

[23] Forkman B, Boissy A, Meunier-Salauen MC, Canali E, Jones RB (2007). A critical review of fear tests used on cattle, pigs, sheep, poultry and horses.. Physiology & Behavior.

[24] Schmid R, Doherr MG, Steiger A (2006). The influence of the breeding method on the behaviour of adult African grey parrots (Psittacus erithacus).. Applied Animal Behaviour Science.

[25] Fox RA, Millam JR (2004). The effect of early environment on neophobia in orange-winged Amazon parrots (Amazona amazonica).. Applied Animal Behaviour Science.

[26] Meehan CL, Mench JA (2002). Environmental enrichment affects the fear and exploratory responses to novelty of young Amazon parrots.. Applied Animal Behaviour Science.

[27] Groothuis TGG, Carere C (2005). Avian personalities: characterization and epigenesis.. Neuroscience and Biobehavioral Reviews.

[28] Greenberg R (1983). The role of neophobia in determining the degree of foraging specialization in some migrant warblers.. American Naturalist.

[29] Misslin R, Cigrang M (1986). Does neophobia necessarily imply fear or anxiety?. Behavioural Processes.

[30] Newman JA, Bergelson J, Grafen A (1997). Blocking factors and hypothesis tests in ecology: Is your statistics text wrong?. Ecology.

[31] Heiblum R, Aizenstein O, Gvaryahu G, Voet H, Robinzon B (1998). Tonic immobility and open field responses in domestic fowl chicks during the first week of life.. Applied Animal Behaviour Science.

[32] Jones RB (1982). Effects of early environmental enrichment upon open-field behavior and timidity in the domestic chick.. Developmental Psychobiology.

[33] Maddocks SA, Goldsmith AR, Cuthill IC (2002). Behavioural and physiological effects of absence of ultraviolet wavelengths on European starlings Sturnus vulgaris.. Journal of Avian Biology.

[34] Garamszegi LZ, Eens M, Janos T (2009). Behavioural syndromes and trappability in free-living collared flycatchers, Ficedula albicollis.. Animal Behaviour.

[35] Apfelbeck B, Raess M (2008). Behavioural and hormonal effects of social isolation and neophobia in a gregarious bird species, the European starling (Sturnus vulgaris).. Hormones and Behavior.

[36] Aulich D (1976). Escape versus exploratory activity - interpretation of rats behavior in open-field and a light-dark preference test.. Behavioural Processes.

[37] Welker WI (1957). “Free” versus “forced” exploration of a novel situation by rats.. Psychological Reports.

[38] Osborne GL (1977). Differences in locomotor activity between rats and gerbils in response to novelty.. Behavioral Biology.

[39] Whishaw IQ, Gharbawie OA, Clark BJ, Lehmann H (2006). The exploratory behavior of rats in an open environment optimizes security.. Behavioural Brain Research.

[40] Belzung C, Lepape G (1994). Comparison of different behavioral-test situations used in psychopharmacology for measurement of anxiety.. Physiology & Behavior.

[41] Wemelsfelder F, Haskell M, Mendl MT, Calvert S, Lawrence AB (2000). Diversity of behaviour during novel object tests is reduced in pigs housed in substrate-impoverished conditions.. Animal Behaviour.

[42] Madruga C, Xavier LL, Achaval M, Sanvitto GL, Lucion AB (2006). Early handling, but not maternal separation, decreases emotional responses in two paradigms of fear without changes in mesolimbic dopamine.. Behavioural Brain Research.

[43] Meerlo P, Horvath KM, Nagy GM, Bohus B, Koolhaas JM (1999). The influence of postnatal handling on adult neuroendocrine and behavioural stress reactivity.. Journal of Neuroendocrinology.

[44] Vallee M, Mayo W, Dellu F, LeMoal M, Simon H (1997). Prenatal stress induces high anxiety and postnatal handling induces low anxiety in adult offspring: Correlation with stress-induced corticosterone secretion.. Journal of Neuroscience.

[45] Aengus WL, Millam JR (1999). Taming parent-reared orange-winged Amazon parrots by neonatal handling.. Zoo Biology.

[46] Barnett SA, Dickson RG, Marples TG, Radha E (1978). Sequences of feeding, sampling and exploration by wild and laboratory rats.. Behavioural Processes.

[47] Holmes A, Parmigiani S, Ferrari PF, Palanza P, Rodgers RJ (2000). Behavioral profile of wild mice in the elevated plus-maze test for anxiety.. Physiology & Behavior.

[48] Macrì S, Würbel H (2006). Developmental plasticity of HPA and fear responses in rats: A critical review of the maternal mediation hypothesis.. Hormones and Behavior.

[49] Francis DD, Diorio J, Plotsky PM, Meaney MJ (2002). Environmental Enrichment Reverses the Effects of Maternal Separation on Stress Reactivity.. J Neurosci.

[50] Sih A, Bell AM, Johnson JC, Ziemba RE (2004). Behavioral syndromes: An integrative overview.. Quarterly Review of Biology.

[51] Dingemanse NJ, Reale D (2005). Natural selection and animal personality.. Behaviour.

[52] Herborn KA, Macleod R, Miles WTS, Schofield ANB, Alexander L (2010). Personality in captivity reflects personality in the wild.. Animal Behaviour.

[53] Mettke-Hofmann C, Ebert C, Schmidt T, Steiger S, Stieb S (2005). Personality traits in resident and migratory warbler species.. Behaviour.

[54] Miller KA, Garner JP, Mench JA (2006). Is fearfulness a trait that can be measured with behavioural tests? A validation of four fear tests for Japanese quail.. Animal Behaviour.

[55] Coleman K, Wilson DS (1998). Shyness and boldness in pumpkinseed sunfish: individual differences are context-specific.. Animal Behaviour.

[56] Bateson M, Matheson SM (2007). Performance on a categorisation task suggests that removal of environmental enrichment induces ‘pessimism’ in captive European starlings (Sturnus vulgaris).. Animal Welfare.

