# Supplementary figures and images for: Fear and Exploration in European Starlings (Sturnus vulgaris): A Comparison of Hand-Reared and Wild-Caught Birds
Source: PLoS One. 2011 Apr 15;6(4):e19074. doi: 10.1371/journal.pone.0019074 (PMC3078136; doi:10.1371/journal.pone.0019074)

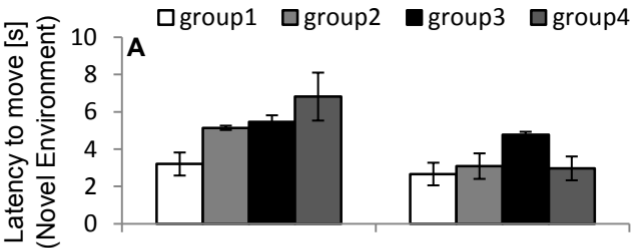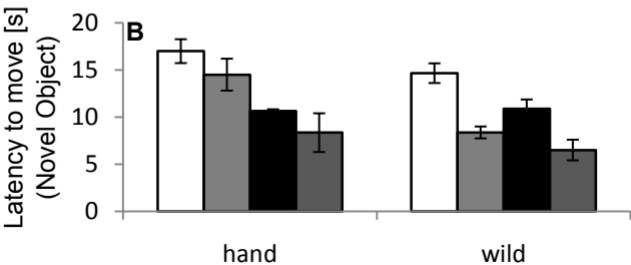

Supplement: Figure S1 — Effect of replicate group. Effect of origin (hand = hand-reared; wild = wild-caught) and replicate group (different colours indicate replicate groups 1 to 4) on latency to move in (A) Novel Environment Test and (B) Novel Object Test 1. Data show group means ± 1 SEM. (PDF) [file pone.0019074.s001.pdf]
